# Treatment of Dislocations of the Head of the Humerus

**Published:** 1884-04

**Authors:** Hal. C. Wyman

**Affiliations:** Prof. Physiology, Mich. College of Med., Detroit


### TREATMENT OF OLD DISLOCATIONS OF THE HEAD OF THE HUMERUS.*

[Detroit Lancet.]

BY HAL C. WYMAN, M.D., PROF. PHYSIOLOGY, MICH. COLLEGE OF MED., DETROIT.

The frequency of dislocations of the shoulder being overlooked until such a time has elapsed that the dislocated parts have contracted from adhesions in unnatural localities, makes it an interesting study to determine the best treatment to pursue when such a time has elapsed after the receipt of injury, that ordinary manipulations do not suffice to restore the head of the humerus to its natural position.

It is impossible for the surgeon to estimate the degree of strength and resisting power exercised by the tissues involved in a dislocation of the shoulder, with anything like the certainty that the engineer calculates the strain which the materials of a bridge will endure. Therefore, there is much left to the judgment of the surgeon which cannot be gauged by fixed rules for determining the amount of force which may be applied with safety in attempts at reducing old dislocations. The fact, which I hope to show by citations from well known writers on dislocations, that in attempting to reduce an old dislocation the arm is put in jeopardy, which often culminates in amputation as a result of injuries inflicted on the great vessels of the arm and axillary region, inclines one to the opinion that a surgeon should have in mind a sufficiently accurate estimate of the force he is using to enable him to avoid inflicting such injuries as will destroy the integrity of blood-vessels and make amputation imperative. All cases which cannot be reduced without jeopardizing the arm should be treated by excision of the head of the humerus, incision of the capsule of the joint or tenotomy of several of the large muscles inserted into the upper end of the humerus, that is, if the case require any surgical treatment. Since it is impossible to determine mathematically what a judicious amount of force is in a given case, it is apparent that our opinions of a quantity which is purely theoretical, must be derived from the experience of others, and the exercise of that peculiar quality of mind known as judgment, a factor in "ordinary skill."

* A paper before the Wayne County Medical Society.

A man of good robust health suffered a dislocation of the shoulder. His medical attendant failed to reduce the dislocation, for causes probably known to himself. Six weeks after the receipt of the injury he went to the clinic at the University of Michigan. Attempts were then made to reduce the dislocation by breaking up the adhesions. The heel of the operator was used as a fulcrum in the axilla while the humerus and arm were used as a lever. The dislocation was not reduced, the attempt failed. Extensive ecchymosis was noticed in the axilla, side and arm soon after the trial at reduction. The patient left the University hospital and went to his home at Rockwood, Michigan. Gangrene soon appeared in the dislocated member, and a surgeon of Monroe, Michigan, amputated the limb near the elbow. This patient threatens the man who first treated the dislocated shoulder with a suit at law for recovery of damages resulting from unskillful treatment. Cases of this kind may well deserve the most thorough study from surgeons. Since the Bar of Michigan permits its members to deal with their clients on the "no cure, no pay" principle, and the lawyer who takes cases "on shares" can lose nothing but his time—a something to him of very small value—in case the jury fails to *salt* the defendant in heavy damages, we may expect frequent prosecutions for malpractice, be our services never so skillfully rendered. An empty sleeve or a crutch is, in the eyes of some lawyers, sufficient cause to drag the poor doctor into court to defend his reputation and his property. Medical men must be prepared to cope with suits at law which are often monstrously unjustifiable. The court usually charges the jury that all that is expected of the doctor is the exercise of ordinary skill, and, that by the term ordinary skill, he means the use of such skill as is ordinarily or commonly used by the doctors in the locality where the defendant lives, and such skill as he is commonly reputed to possess. The surgeon on the frontier with the rude and meager means at his command in the treatment of injuries, would not be held by the court to the same degree of responsibility with the hospital surgeon, who has all the advantages of counsel and approved apparatus at his command. The more distinguished the surgeon is, the greater his responsibility. Nothing in surgery is better calculated to show the above implied varieties of ordinary skill than the cases of old dislocation of the shoulder.

Bearing upon this subject, Clark, in his "Manual of Surgery," says: "After the lapse of three months it is almost hopeless to attempt the reduction of a dislocated shoulder. If the patient has begun to move his arm, and a false joint has in some manner been established, the surgeon had better not interfere. The most serious, and even fatal, consequences may follow an injudicious attempt at reduction."

Ashurst in his "International Encyclopedia of Surgery" says: "A majority of dislocated shoulders become incurable after twelve weeks. Jarvis's adjuster is so essential an instrument, that little success or safety can be expected without it, save in cases not very difficult. The danger attending efforts to reduce old dislocations is a constant factor, and should be explained to patients, before submitting to this operation."

Besides the fracture of the surgical neck of humerus, and even of a rib have been known to take place in the violent efforts at reduction, and also there is constantly to be feared a rupture of the axillary artery or vein, accidents which usually prove fatal. In one case this resulted from a boot heel used under the arm in counter extension. In the same manner the innervation of the parts below the axilla has been injured. These accidents have occurred most frequently in the practice of most eminent surgeons. In one remarkable case the surgeon tore an arm completely from the side of an aged woman. He then goes on to relate the case of an old farmer who, after four years from the time the arm was dislocated, and which an eminent surgeon had pronounced hopeless, was cured by a horse rearing, when its halter happened to be fastened to the dislocated arm. Further on he says:

"I believe subcutaneous section to be devoid of danger, when performed with full antiseptic precautions, and recommend it always rather than leave the shoulder hopelessly maimed. No case has ever come under my care in which this operation has been required, but I should certainly resort to it rather than leave the shoulder unreduced, and in preference to resection." (Subcutaneous osteotomy has been successfully performed by Dr. Mears, of Philadelphia, in a case of old, unreduced dislocation of shoulder.)

"Resection of the head of the humerus must be performed in cases where the axillary plexus is so compressed as to cause paralysis, and where other means have failed."

Speaking on this subject, Chelius, in his "System of Surgery," says: "In the reduction of dislocations of several weeks' standing, the arm must be forcibly moved in every direction, to break up new adhesions. Weinhold cut through the tendon of the pectoralis major, because it did not yield in an old dislocation. Old dislocations may often be satisfactorily reduced, but the attempts must not be carried too far, as dangerous symptoms may ensue."

Gibson noticed the rupture of the axillary artery in reducing an old dislocation.

Dieffenbach, while making extension and counter-extension, divided the tendons of pectoralis major and latissimus dorsi, the teres major and minor, and the false ligaments surrounding the new joint, when, with the extension made, the head suddenly slipped back into its socket.

Erichsen, in his "Science and Art of Surgery," says: "That the accidents that have occurred in the reduction of old dislocations are due to the use of too much force in breaking up of new adhesions, or from pathological changes in limb itself. Among the first are: laceration and bruising of skin. subcutaneous areolar tissue and muscles, with extravasation of blood; also fracture of humerus, laceration of axillary vessels and nerves, and evulsion of limb."

"In the fracture of the humerus, the neck usually gives way in bringing the arm across the chest, when the shaft becomes exposed to fracture by pressure in a transverse direction. This accident is of frequent occurrence. It has happened to Petit, Pott, Larrey, Birard, Denonvilliers and others."

"Fracture of the ribs by pressure against the walls of the chest is supposed to have occurred in some cases."

"Rupture of one of the large blood-vessels in the axillary region sometimes occurs. One case is reported to have been caused by the pressure of a boot heel. Others by the humerus having become adherent to the blood-vessel, and lacerating this when torn away. The instances on record of laceration of axillary artery, and consequent traumatic aneurism in the axilla. are very numerous, and are a warning to the surgeon not to use too much force."

"In Dupuytren's case, in the reduction of a dislocation of six weeks' standing, after two or three months had elapsed from the time of reduction, a tumor appeared under the arm. This was mistaken for an abscess and opened. The patient died a week after from secondary bleeding."

A singular case is related of Nelaton, who was compelled to tie the subclavian.

"Pelletan made a similar fatal mistake to Dupuytren's. So also with cases reported by Verdue, Petit, Platner and Laudet. Sir C. Bell also reports a case similar, in which amputation became necessary. In four cases the subclavian had been ligatured. These happened two to Gibson, one to Blackman, and one to Warren. One only, Warren's, was successful.

" In one case related by Frink, the patient, a female 26 years old, died within an hour and a half after reduction had been performed. Post mortem showed laceration of axillary vein.

" Injuries to axillary nerve causing paralysis have also been related. One case is related by Bilroth, who after reducing a dislocation of nine months' standing, and which had become partially paralyzed, found after the reduction that total paralysis had taken place.

" Besides this, sudden death from syncope and exhaustion have frequently followed the reduction of old cases of dislocation. Guerin had a case of a woman in which evulsion of elbow joint followed the reduction. In this case no extra force seems to have been used.

" In cases of old standing, where symptoms of pressure on the large nerves and vessels are present, and which may be injured by an attempt at reduction of the bone, Bilroth recommends excision of its head. This has been successfully done by Langenbeck in a case of paralysis from pressure. "

Cooper in his Dictionary of Surgery says, " The common advice in such cases is that no attempt at reduction be made, as it would be useless in regard to the dislocation, and might be injurious to the patient from the excessive stretching of the parts. The first attempts frequently fail, and the dislocated head of the bone continues unmoved, notwithstanding the most violent efforts.

" Notwithstanding the encouragement given by Desault to making attempts to reduce old dislocations of the humerus, experience proves, that when the bone has been out of its place more than a month, success is rarely obtained. And as for the danger from long protracted, immoderate force, cases which have been cited prove that caution is here a virtue which cannot be too highly recommended. Another instance, in which a woman died from the violence used in extension, is reported by Sir Ashley Cooper."

Liston in his " Elements of Surgery, " says: " In case of old dislocations, where the movement of the bone favors the supposition that it has found a new cavity, and the glenoid cavity has from disease become changed, the patient will most probably be put to a great deal of pain, without experiencing improvement of the limb, indeed the movement and power may prove less than before. In old men the force sufficient for the reduction cannot be employed without great risk of laceration of nerves, blood-vessels, and muscles. No standard can be fixed for the degree of force that is necessary and safe. The surgeon may be foiled, even after the most powerful efforts, in the reduction of a dislocation of only two or three weeks' standing, whilst, by the use of but slight force, he may succeed in one of as many months."

Sir B. Bell in his "System of Surgery" says: "In dislocations of long continuance, the most expert practitioners often fail; for in such cases the head of the bone has often formed a new socket in the contiguous parts, from whence it cannot be dislodged without tearing asunder some of the muscles with which it is surrounded; and when dislodged our efforts may be rendered abortive, by the cavity in which the bone should be lodged being diminished. In all cases, therefore, of long duration, that is where the bone has been out of its socket six months or more, although it may be proper to make some attempts to replace the dislocated bones, yet none that require much force should be persisted in, for the attempt must always be of uncertain success, while it necessarily gives much pain, at the same time that it is apt to render the motion of the head in its artificial socket, which it generally forms for itself, more stiff than it was before." Frank Hamilton, speaking of old dislocations of shoulder, says: "I make it my maxim never to attempt to accomplish by complicated and violent measures what may be done as well by more simple and gentle means."

Agnew in his "Surgery" says: "If the bone has been out of place for several months, and is moderately movable in its new position; if following the injury there has been a history of severe pain, of inflammation, and symptoms of union between the blood-vessels and plexus of nerves and the head of the bone; and if no severe pain is any longer experienced in movement of the arm. and there is moderate use of it, the surgeon will do well not to urge reduction.

"If, however, the patient experiences great pain, rendering the limb quite helpless, and if he insists on efforts being made to replace the bone, after fully understanding the dangers incurred, the surgeon will be justified in making the attempt, never, however, presuming to transcend what, in his own judgment, is the limit of safety, in the use of measures which he may employ for the purpose of reduction.

"Should all other measures fail, there remains the operation of Dr. Mears, that of dividing by means of Adam's saw, subcutaneously, the surgical neck of the humerus, and establishing a false joint.

"The accidents that may occur during the employment of forcible measures are laceration of muscles, rupture of blood-vessels, and injury to nerves.

"An instance of accident of first class occurred in St. Bartholomew Hospital during an attempt to restore a dislocation of eight weeks' standing, in which the pectoral muscles were torn, and the axilla laid open, although no force was used other than that employed by a house surgeon pulling on the arm, and having his unbooted foot in the axilla. The muscles in this case, however, were in a state of degeneration.

"There are extravasations of blood met with, after the use of forcible efforts at reduction, which came from comparatively small vessels, chiefly veins.

"The axillary plexus of nerves may be injured in forcible attempts to reduce old cases of dislocations, causing paralysis of the entire arm, or of certain groups of muscles.

"In several cases of attempted forcible reduction of old cases of dislocations, fracture of the humerus has taken place. Hamilton notices three cases of this complication. The same accident happened in the author's own experience, in the Pennsylvania Hospital, before a class of students. In the same institute and before a similar audience, occurred the same accident, under the hands of Dr. Hunt.

"The inflammation which ensues from the forcible disruption of the adhesions between the head of the bone and the tissues of the axilla, may be followed by an abscess. Mr. Hutchinson, of London, lost a patient from this cause."

Holmes in his "System of Surgery" says: "In cases that have been neglected, it often becomes a question as to whether we are justified in attempting a reduction, or whether the head of the bone should be left, with the new connections it has formed, undisturbed. On this point the surgeon must exercise his own judgment in each particular case; but, as a general rule, the limit laid down by Sir Ashley Cooper, of twelve weeks, may be taken as a guide.

"Many surgeons have, indeed, ventured further, and cases are reported of successful reductions after twelve and even eighteen months. But it must not be forgotten that we know less than we should do of the serious accidents which have often attended such attempts, one of the most frequent of which is laceration of the axillary artery. Already a considerable list of fatal cases from this cause might be collected."

A case of this kind was published in the second volume of St.

Bartholomew's Hospital Reports of 1866, page 96: A gardener fell from a roof to the ground and dislocated the left humerus. The dislocation was reduced, but was afterwards reproduced by an incautious movement of the arm. At the end of the sixteenth week he was sent to the hospital, where the head of the humerus was plainly felt in the axilla, drawn up toward the coracoid process. It was reduced, under chloroform, with the use of very slight force in circumducting the arm. Directly afterwards a swelling rapidly rising and projecting the pectoral muscle was seen, and as it did not pulsate, and the radial pulse beat naturally, it was thought that the swelling might be due to the rupture of a vein or a muscular artery. As the swelling continued to increase, and the limb was greatly ecchymosed, it was freely laid open, and after turning out a large quantity of clot, a stream of arterial blood was seen to issue from the upper wall of the axillary artery. The vessel was ligated above and below the orifice. Gangrene of the limb ensued, and the man died with symptoms of pulmonary embolism.

I think I have cited a sufficient number of opinions to show that great caution should always be exercised in attempts at reduction of old dislocations of the shoulder. Many of these old dislocations have developed quite useful members, notwithstanding the unnatural relations of the articulations; but there are other cases in which the deformity is the source of such severe pain that life becomes a burden and the patient gladly consents to any measures which the surgeon may propose for his relief.

It becomes then a matter of no mean responsibility to decide upon the best plan of treatment to pursue. While I believe, after a careful review of the literature of old dislocations and a serious study of the anatomy and physiology of the tissues concerned in any old displacement of the shoulder joint, that the best method of treatment may be understood by the terms tenotomy, incision and resection, I have reference only to such cases as make some treatment necessary by the physical suffering which they inflict, and are not amenable to judicious manipulation. It is apparent that the important question to decide is what is a judicious attempt at reduction by breaking down adhesions. I have stated that it is impossible to know with mathematical precision the amount of resistance the structure involved in a dislocation may offer without breaking under the manipulations of an operator. So may it be said of all the operations of surgery, but there is a well defined knowledge, the result of experience in handling living animal tissues known as

*tactus eruditus*, or delicacy of touch, etc., and which is directly the opposite of what is known as rough handling, bungling surgery, etc. It is a conscious appreciation of the nice distinctions between these two extremes of manipulation which is the guide to a judicious use of force in the treatment of old dislocations. The surgeon who has the interest of his patient and good surgery at heart will not push his efforts at reduction by manipulation beyond this limit. After it has been reached and the humerus still retains its artificial position, the knife and perhaps the saw should be resorted to.

